# The miR‐124‐3p regulates the allergic airway inflammation and remodeling in an ovalbumin‐asthmatic mouse model by inhibiting S100A4

**DOI:** 10.1002/iid3.730

**Published:** 2023-02-03

**Authors:** Min Liu, Shuang Liu, Fajiu Li, Chenghong Li, Shi Chen, Xiaoyan Gao, Xiaojiang Wang

**Affiliations:** ^1^ Department of Pulmonary and Critical Care Medicine Affiliated Hospital of Jianghan University Wuhan Hubei P.R. China

**Keywords:** airway remodeling, asthma, epithelial–mesenchymal transition, miR‐124‐3p, S100A4, TGF‐β1/smad2 signaling pathway

## Abstract

**Objective:**

Asthma is a chronic respiratory disease with an increasing incidence every year. microRNAs (miRNAs) have been demonstrated to have implications for asthma. However, limited information is available regarding the effect of miR‐124‐3p on this disease. Therefore, this study aimed to explore the possible effects of miR‐124‐3p and S100A4 on inflammation and epithelial–mesenchymal transition (EMT) in asthma using mouse models.

**Method:**

Ovalbumin was used to induce asthmatic mouse models. Lung injury in mouse models was assessed, and the bronchoalveolar lavage fluid of mice was collected to determine the number of eosinophilic granulocytes and assess inflammation. The expression levels of miR‐124‐3p, S100A4, E‐cadherin, N‐cadherin, Snail1, vimentin, and TGF‐β1/Smad2 signaling pathway‐related proteins were measured using reverse transcription‐quantitative polymerase chain reaction (RT‐qPCR) and western blot analysis. In vitro experiments, cells were transfected with miR‐124‐3p mimics or inhibitors to test the expression of S100A4 by RT‐qPCR and western blot analysis, and the mutual binding of miR‐124‐3p and S100A4 was validated by dual‐luciferase reporter gene assay.

**Results:**

Overexpression of miR‐124‐3p or inhibition of S100A4 expression attenuated bronchial mucus secretion and collagenous fibers and suppressed inflammatory cell infiltration. Additionally, upon miR‐124‐3p overexpression or S100A4 suppression, eosinophilic granulocytes were decreased, interleukin‐4 (IL‐4) and IL‐13 expression levels were reduced in the bronchoalveolar lavage fluid, serum total IgE level was reduced, and the TGF‐β1/Smad2 signaling pathway was suppressed. Mechanically, a dual‐luciferase reporter gene assay verified the binding relationship between miR‐124‐3p and S100A4.

**Conclusion:**

miR‐124‐3p can negatively target S100A4 to attenuate inflammation in asthmatic mouse models by suppressing the EMT process and the TGF‐β/smad2 signaling pathway.

## Graphical abstract

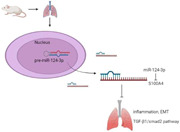

## 1 INTRODUCTION

Asthma, a chronic respiratory disease, is associated with an increasing incidence and reduced lung function.^1^ Airway inflammation and hyper‐responsiveness are the two major characteristics of asthma, and this disease involves various cell types and the activation of inflammatory genes.^2^ Generally, the airway epithelium is responsible for the management of inflammatory and immune responses to inhaled environmental insults, including allergens, viruses, and pollutants.^3^ Airway remodeling, which is as important as inflammation, is believed to have a certain relationship with asthma pathogenesis.^4^ Airway remodeling in asthma requires long‐term inflammatory cell infiltration, which is characterized by an increased number of eosinophilic granulocytes and CD4+ T cells in the airways.^5^ Airway remodeling, mainly driven by chronic airway inflammation, is a major cause of refractory asthma, which can accelerate subepithelial fibrosis by enhancing epithelial–mesenchymal transition (EMT).^6^ EMT is the underlying reason for fibroblasts, goblet cells, and pneumocyte hyperplasia in diverse lung diseases, which leads to lung fibrosis.^7^ The plastic and dynamic airway epithelium has been indicated to result in airway remodeling via EMT in asthma.^8^ More studies are being conducted to explore the pathogenesis of asthma; however, its prognosis is still far from satisfactory, and no curative therapy is currently available.

MicroRNAs (miRNAs) are associated with chronic inflammatory and aspiratory diseases.^9^ Many miRNAs have been reported to have implications for pulmonary injuries. For example, the downregulation of miR‐138‐5p expression induced by metformin treatment was reported to suppress the MAPK pathway and therefore alleviate acute respiratory distress syndrome.^10^ Further, in traumatic acute lung injury, exosomal miR‐124‐3p in mesenchymal stem cells can improve oxidative stress injury and suppress inflammatory responses.^11^ Among those miRNAs, miR‐124‐3p is closely related to the progression of inflammation‐related diseases and carcinomas. For example, miR‐124‐3p, regulated by SNHG16, has been reported to regulate the EMT and proliferation of colorectal cancer cells through downstream MCP‐1.^12^ In addition, miR‐124‐3p was proven to be associated with macrophage polarization in lung cancer^13^ and to suppress pulmonary metastasis in osteosarcoma models.^14^ Liu et al.^15^ have supported that miR‐124‐3p might relieve Type 2 inflammation in allergic rhinitis (AR) by modulating the interleukin‐4Rα (IL‐4Rα) signaling. Moreover, upregulation of miR‐124‐3p has been suggested to relieve pathological changes, decrease the frequency of nasal rubbing and sneezing, and apoptosis of nasal mucosa.^16^ However, limited information is available regarding the effect of miR‐124‐3p on the inflammatory response and EMT process in asthma.

S100A4 is an inflammation‐related protein that can be found in inflammatory sites of various tissues.^17^ S100A4 expression was found to be upregulated in asthmatic mouse models, and evidence supports its involvement in the pathogenesis of this disease.^18^ One study that explored the effect of miR‐124‐3p on neointimal proliferation identified a regulatory effect of miR‐124‐3p on S100A4.^19^ Meanwhile, another study also revealed a binding relation between miR‐124 and S100A4 in the proliferation and migration of vascular smooth muscle cells.^19^ Accordingly, in this study, online software predicted the interaction between miR‐124‐3p and S100A4. Nevertheless, the functions of the miR‐124‐3p/S100A4 axis in the ovalbumin (OVA)‐asthma model are still in their infancy. Therefore, in this study, we aimed to explore the possible effects of miR‐124‐3p and S100A4 on inflammation and EMT in asthma using mouse models, and we speculated that miR‐124‐3p might play a role in asthma by regulating S100A4 expression.

## 2 MATERIALS AND METHODS

### 2.1 Asthmatic mouse models

Forty‐two BALB/c mice (6‐week‐old, male) were randomly classified into the following groups: sham group (control for Model group), Model group (OVA‐induced asthmatic mice), lentivirus (LV)‐negative control (NC) group (control for LV‐miR‐124‐3p group), LV‐miR‐124‐3p group (asthmatic mice with the overexpression of miR‐124‐3p), LV‐sh‐NC group (control for LV‐sh‐S100A4 group), and LV‐sh‐S100A4 group (asthmatic mice with the downregulation of S100A4 expression). Six mice were used for each group.

*Sensitization* ^20^: Mice, except those in the sham group (receiving an equal volume of phosphate‐buffered saline [PBS]), were injected intraperitoneally with 40 μg of OVA (O1641, Sigma) and 2 mg PBS (Hyclone) containing 10% aluminum hydroxide (Aladdin) on Days 0, 7, and 14.

*Stimulation*: Between Day 21 and Day 55, mice, except those in the sham group, were sensitized with 20 min of the atomized inhalation of PBS solution containing OVA (5%). After inhalation, 20 μl of OVA (40 mg/ml) was given to mice through nasal inhalation, three times per week.

*Therapy*: From Day 26, mice, except those in the sham group, received 100 μl of normal saline via tail vein injection once per week, containing 5 × 10^8^ TU/ml lentivirus, miR‐124‐3p lentivirus (LV‐miR‐124‐3p), lentivirus to inhibit S100A4 expression, or LV‐NC/LV‐sh‐NC.^21^ All lentiviruses were obtained from GeneChem. On Day 55, the mice were euthanized, and their lungs were collected.^22^ The design of the experiment was approved by the ethical committee of the Affiliated Hospital of Jianghan University. The experiments were performed according to the ARRIVE guidelines.

The animals were euthanized as follows: Cervical dislocation was performed under deep anesthesia (intraperitoneal injection of 100 mg/kg ketamine [K] + 10 mg/kg xylazine [X]). The right lung tissues were collected and placed in a cryopreservation tube, and then stored in a liquid nitrogen tank. The left lung tissues were fixed with 10% formaldehyde and embedded in paraffin.

### 2.2 Analysis of bronchoalveolar lavage fluid (BALF)

**Figure 1:**
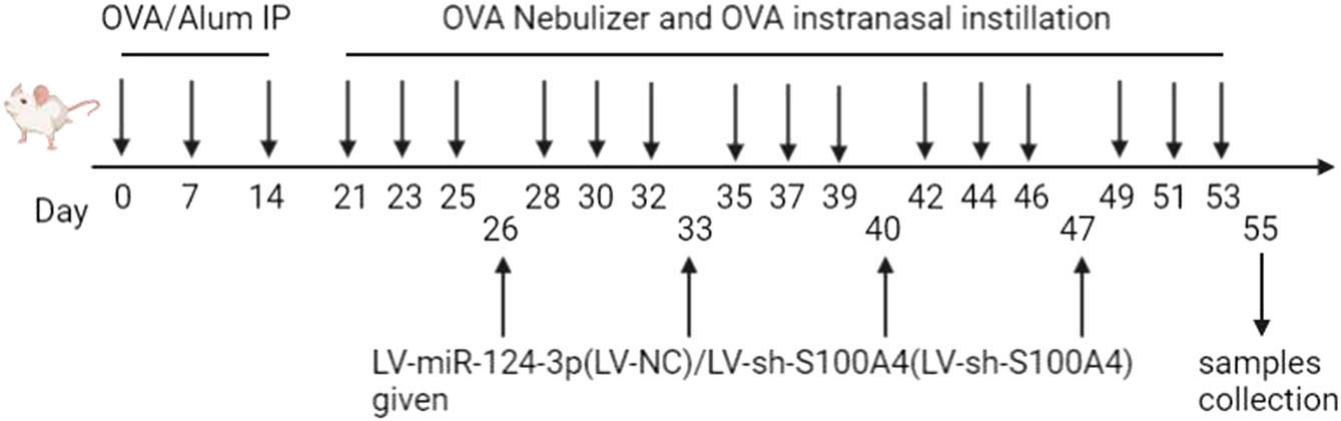
Experimental flow chart. OVA, ovalbumin.

After anesthesia, the neck skin of mice was incised, and the subcutaneous tissues were separated to expose the trachea. Subsequently, the left bronchus of the mice was ligated, a venipuncture trocar was placed in the right bronchus, and ice‐cold PBS (0.5 ml) was instilled into the right lung, followed by withdrawal. Liquid volume ≥80% was considered to be qualified, and this operation was repeated three times. Next, the BALF was collected and centrifuged to precipitate the cells. The supernatant was collected and stored at 80°C, whereas cell precipitates were resuspended in PBS (1 ml); inflammatory cells were counted with a hemocytometer, and eosinophils were counted after Wright (32857, Sigma) and Giemsa (48900, Sigma) staining. The experimental flow chart is presented in Figure 1.

### 2.3 Enzyme‐linked immunosorbent assay (ELISA) for cytokine expression

The lung tissues were ground and homogenized. An ELISA kit was used for the detection of IL‐4 (PI612; Beyotime) and IL‐13 (P5948; Beyotime). Additionally, the serum from each group of mice was collected to examine the total immunoglobulin E (IgE) content (PI476, Beyotime) in serum under the instructions of the kit.

### 2.4 Hematoxylin and eosin staining

The lung section (5 μm) were de‐waxed in xylene and washed five times in gradient ethanol (Aladdin) before hematoxylin (2 g/L, Aladdin) staining for 5 min. The sections were immersed in a hydrochloric acid–ethanol solution (Aladdin) for 30 s and then immersed in a 1% eosin solution (Aladdin) for 2 min before dehydration in absolute ethanol (Aladdin) and sealing with neutral resins. The morphology of the cells was observed and photographed under a microscope (CX‐21, Olympus) with a micropublisher system (200× and 400×).

### 2.5 Periodic acid‐Schiff (PAS) staining

After washing in distilled water, the slices were rinsed in periodic acid solution (10 min), washed with running water (10 min), immersed in Schiff solution (10 min), and washed with running water (5 min). The slices were stained with hematoxylin (3 min), washed, hydrated, transparentized, and sealed. Images were captured under a microscope (CX‐21) with a micropublisher system (200× and 400×). PAS staining was interpreted using ImageJ to calculate the sedimentation of cells stained red. The PAS staining kit was purchased from Beyotime company.

### 2.6 Masson staining

The collected lung tissues were dewaxed and hydrated before hematoxylin staining for 3 min. Thereafter, the slices were washed and re‐stained with Masson's solution for 3 min before being rinsed in 2% aqueous acetic acid solution, which was followed by differentiation in 1% phosphomolybdic acid solution and staining with toluidine blue. The slices were washed with a 2% aqueous acetic acid solution, hydrated, made transparent, and sealed. The staining was observed under a microscope (CX‐21) with a micropublisher system (200× and 400×). The collagen volume fraction was calculated using ImageJ. The chemical reagents used in Masson staining were purchased from Sinopharm Chemical Reagent Co., Ltd.

### 2.7 Cell culture and transfection

Human normal pulmonary epithelial cells (BEAS‐2B), from the American Type Culture Collection, were cultured in Dulbecco's modified Eagle's medium (11965092, Gibco) containing 10% fetal bovine serum (16140071, Gibco) at 37°C with 5% CO_2_. The miR‐124‐3p mimic, miR‐124‐3p inhibitor, mimic‐NC, and inhibitor‐NC were purchased from Genechem. After transferring cells into a 60 mm culture dish (3.0 × 10^5^ cells per dish, Corning,) for 24 h, they were incubated with Opti‐MEM I reduced serum culture medium (31985062, Gibco) containing 3 μg of plasmid with Lipofectamine 2000 reagent (11668019, Invitrogen) and 8 ng/ml polybrene (TR‐1003, Sigma‐Aldrich) for 48 h.

### 2.8 Dual‐luciferase reporter gene assay

Starbase (http://starbase.sysu.edu.cn/index.php) was used to predict the binding sites of S100A4 for miR‐124‐3p, and the mutant sites were designed accordingly. The wild type (WT‐S100A4) and mutant S100A4 (MT‐S100A4) were cloned into psiCHECK‐2 (C8011, Promega). Next, psiCHECK‐2 was cotransfected with the miR‐124‐3p mimic or mimic‐NC into BEAS‐2B cells. After transfection for 48 h, the Dual‐Glo luciferase reporter analysis system (Promega) was used to detect *Renilla* luciferase and firefly luciferase activities.

### 2.9 Reverse transcription‐quantitative polymerase chain reaction (RT‐qPCR)

Total RNA was extracted using TRIzol reagent (15596018, Invitrogen), and total RNA (1 μg) was converted into cDNA using the Primescript RT Reagent Kit (3733, TaKaRa). Real‐time PCR was conducted using the SYBR Green qPCR master mix kit (330500, Qiagen) and Bio‐Rad CFX384 Touch system (Bio‐Rad). All reactions were performed in triplicate. The average 2^ΔΔCt^ value was calculated using data obtained from three measurements based on the 2^−ΔΔCt^ method. Reverse transcription and real‐time PCR were performed using the All‐in‐One miRNA quantitative reverse transcriptase PCR detection kit (QP010, GeneCopoeia) following the manufacturer's instructions. U6 small nuclear RNA (snRNA) was used as an endogenous control for miRNA. Primer sequences used for RT‐qPCR are listed in Table 1.

**Table 1:** Primer gene sequences for RT‐qPCR

Name of primer	Sequences(5′–3′)
miR‐124‐3p‐F	ccgTaagTggcgcA
miR‐124‐3p‐R	TGGTGTCGTGGAGTCG
S100A4‐F	TCTCAACACACTGTTGGCGT
S100A4‐R	CACAGGCAGGACCCACTTAC
N‐Cadherin‐F	CAACTTGCCAGAAAACTCCAGG
N‐Cadherin‐R	ATGAAACCGGGCTATCTGCTC
E‐Cadherin‐F	CGAGAGCTACACGTTCACGG
E‐Cadherin‐F	GGCCTTTTGACTGTAATCACACC
Snail‐1‐F	ACGAGGTGTGACTAACTAT
Snail‐1‐R	CGACAAGTGACAGCCATT
Vimentin‐F	CCCTTGACATTGAGATTGCCACCT
Vimentin‐R	GTGGGTATCAACCAGAGGGAGTGA
TGF‐β1‐F	CTGTACTCTGGCAGTGACCC
TGF‐β1‐R	CCACGTAGTAGACGATGGGC
Smad2‐F	TCTGCACAGAGTAGGGTGGT
Smad2‐R	TGGACCAAGGCGAAAGGAAA
U6‐F	CTCGCTTCGGCAGCACA
U6‐R	AACGCTTCACGAATTTGCGT
GAPDH‐F	GTGGCTGGCTCAGAAAAAGG
GAPDH‐R	GGGGAGATTCAGTGTGGTGG

Abbreviations: F, forward primer; R, reverse primer

### 2.10 Western blot analysis

The total protein concentration was measured using a BCA protein detection kit (23227, Thermo Fisher Scientific). The proteins, diluted with 5× loading buffer, were electrophoresed with a 12% separating gel for 90 min and then incubated with skimmed milk powder diluted in PBS (at 5% (w/v)) for 1 h to block nonspecific reactions. The cells were then incubated with primary antibodies against S100A4 (1:500, ab124805, Abcam), Smad2 (1:500, ab40855, Abcam), TGF‐β1 (1:500, ab215715, Abcam), N‐cadherin (1:1000, ab76057, Abcam), E‐cadherin (1:1000, ab194982, Abcam), Snail1 (1:1000, ab53519, Abcam), or vimentin (1:1000, ab92547, Abcam) overnight at 4°C. The secondary antibody (1:500, ab150077, Abcam) was then added for incubation for 1 h at room temperature. The images were photographed using a BioSpectrum Imaging System (UVP).

### 2.11 Statistical analyses

Data were analyzed using GraphPad Prism 8.0. All data are expressed as the mean ± standard error of mean (SEM). The distribution of data was verified using the Shapiro–Wilk test. Analysis of variance and Student's *t*‐tests were applied for comparisons among three or more groups and for comparisons between two groups, respectively, with a Bonferroni test for the post hoc analysis. Statistical significance was set at a *p* value < 0.05.

## 3 RESULTS

### 3.1 Altered expression of miR‐124‐3p and S100A4 in asthmatic mouse models

**Figure 2:**
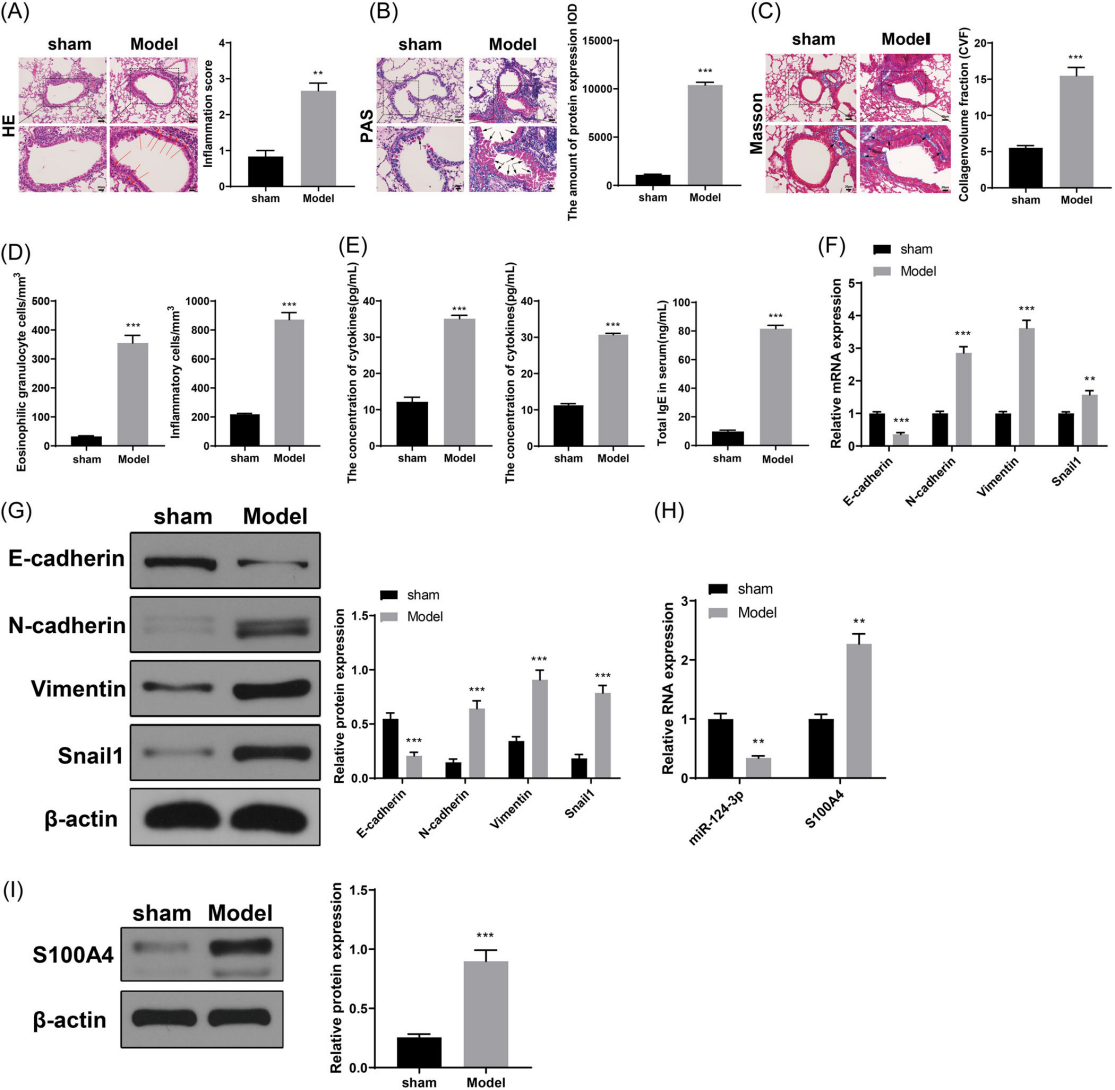
Ovalbumin‐induced asthmatic mouse models had increased expression of S100A4 and suppressed expression of miR‐124‐3p. After OVA treatment, H&E (A), PAS (B), and Masson staining (C) were applied to observe injury in lung tissues. The numbers of eosinophilic granulocytes in BALF were also counted (D). ELISA was utilized to examine the expressions of inflammatory cytokines, and serum total IgE level (E). RT‐qPCR (F) and western blot analysis (G) were implemented to detect the expression levels of E‐cadherin, N‐cadherin, Snail1, and Vimentin. The expression levels of S100A4 and miR‐124‐3p in lung tissues were detected by RT‐qPCR (H) and western blot analysis (I). **p < .01, *** *p* < .001. Each group had six mice and all experiments were conducted for three times. BALF, bronchoalveolar lavage fluid; ELISA, enzyme‐linked immunosorbent assay; EMT, epithelial–mesenchymal transition; H&E, hematoxylin and eosin; OVA, ovalbumin; PAS, periodic acid‐Schiff; RT‐qPCR, reverse transcription‐quantitative polymerase chain reaction.

Mice in the model group showed increased inflammatory cell infiltration in and around the bronchial tubes and vessels, and increased bronchial mucus secretion and collagenous fibers were found in the pulmonary parenchyma and respiratory airway (Figure 2A–C, *p* < .01). BALF analysis showed an increased number of inflammatory cells and eosinophilic granulocytes (Figure 2D, *p* < .01, vs. sham group), along with elevated IL‐4 and IL‐13 expression and serum total IgE level (Figure 2E, *p* < .01, vs. sham group) in the model group. The expression levels of EMT‐related proteins in lung tissues were measured to investigate whether the EMT process is initiated in asthma. Mice in the model group had suppressed expression of E‐cadherin and increased expression of N‐cadherin, Snail1, and vimentin (Figure 2F,G, *p* < .01, vs. sham group). Mice in the model group had increased S100A4 expression and suppressed miR‐124‐3p expression (Figure 2H,I, *p* < .01, vs. sham group).

### 3.2 Overexpression of miR‐124‐3p or knockdown of S100A4 attenuates asthma progression and regulates the EMT process

The RT‐qPCR results showed that mice in the LV‐miR‐124‐3p group had increased miR‐124‐3p expression in lung tissues (Figure 3A, *p* < .01, vs. LV‐NC group), whereas mice in the LV‐sh‐S100A4 group had suppressed expression of S100A4 in lung tissues (Figure 3B,C, *p* < .01, vs. LV‐sh‐NC group). Inflammation and lung injury were also measured in asthmatic mouse models. The LV‐miR‐124‐3p and LV‐sh‐S100A4 groups showed suppressed inflammatory cell infiltration, less bronchial mucus secretion, a decreased amount of collagenous fibers (Figure 3D–F, *p* < .01), fewer inflammatory cells and eosinophilic granulocytes, and reduced secretion of IL‐4 and IL‐13, and serum total IgE level (Figure 3G,H, *p* < .01). The detection of EMT‐related factors showed that the LV‐miR‐124‐3p and LV‐sh‐S100A4 groups had elevated expression of E‐cadherin and suppressed expression levels of N‐cadherin, Snail1, and vimentin (Figure 3I,J, *p* < .01).

Figure 3Asthma in mouse models can be attenuated by overexpression of miR‐124‐3p or suppression of S100A4. (A) RT‐qPCR was performed to detect the expression of miR‐124‐3p in lung tissues; RT‐qPCR (B) and western blot analysis (C) were performed to measure the S100A4 mRNA and protein expression levels. The lung injury of asthmatic mouse models was assessed by H&E (D), PAS (E), and Masson staining (F). The cell number of eosinophilic granulocytes in BALF was assessed (G). ELISA was performed to detect the expression levels of inflammatory IL‐4 and IL‐13 in BALF, and serum total IgE level (H). The expressions of EMT‐related proteins, E‐cadherin, N‐cadherin, Snail1, and Vimentin in lung tissues were measured by RT‐qPCR (I) and western blot analysis (J). **p < .01, ***p < .001.  Each group had six mice and all experiments were conducted for three times. BALF, bronchoalveolar lavage fluid; ELISA, enzyme‐linked immunosorbent assay; EMT, epithelial–mesenchymal transition; H&E, hematoxylin and eosin; IL, interleukin; PAS, periodic acid‐Schiff; RT‐qPCR, reverse transcription‐quantitative polymerase chain reaction.

**Figure:**
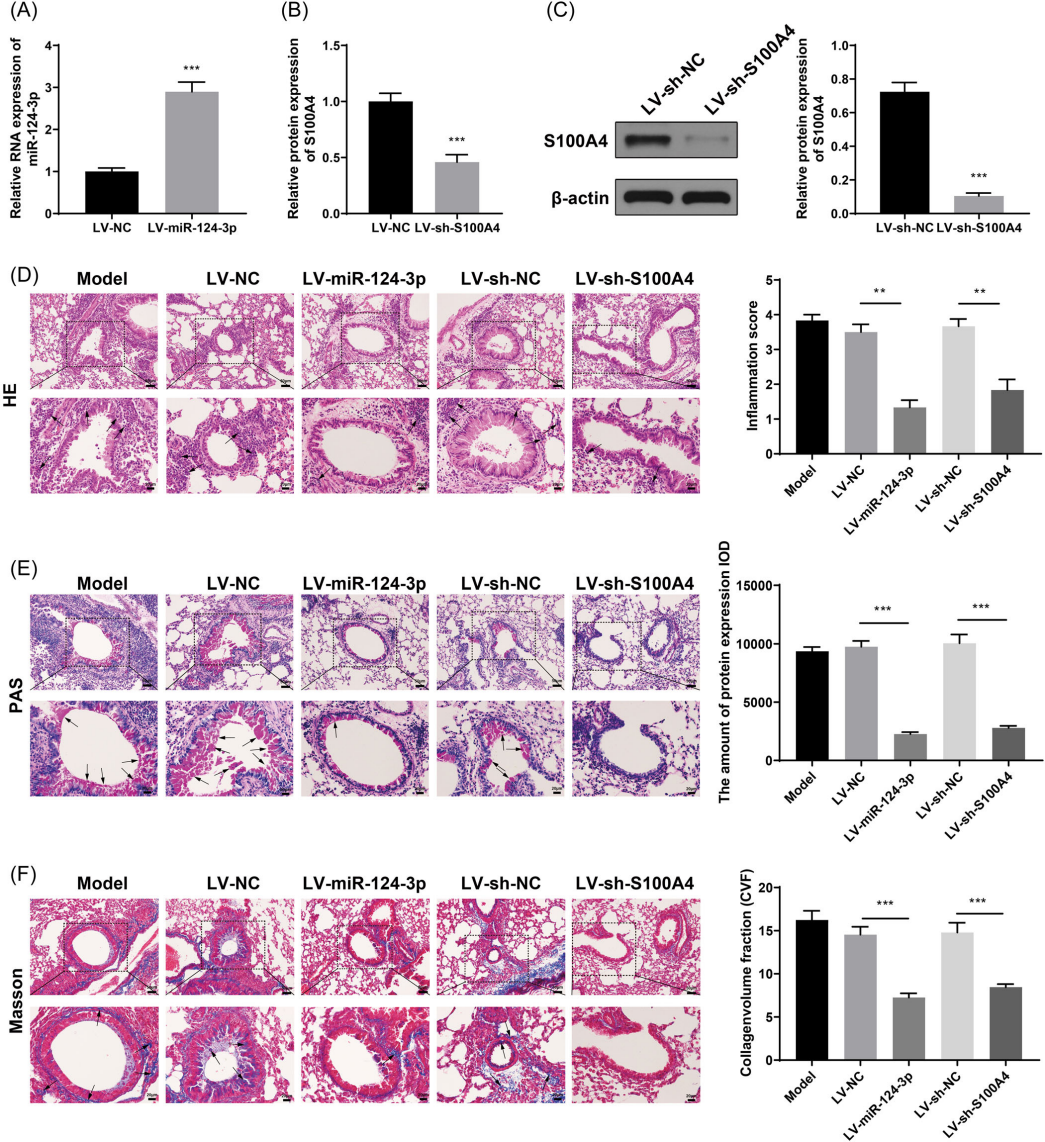

**Figure:**
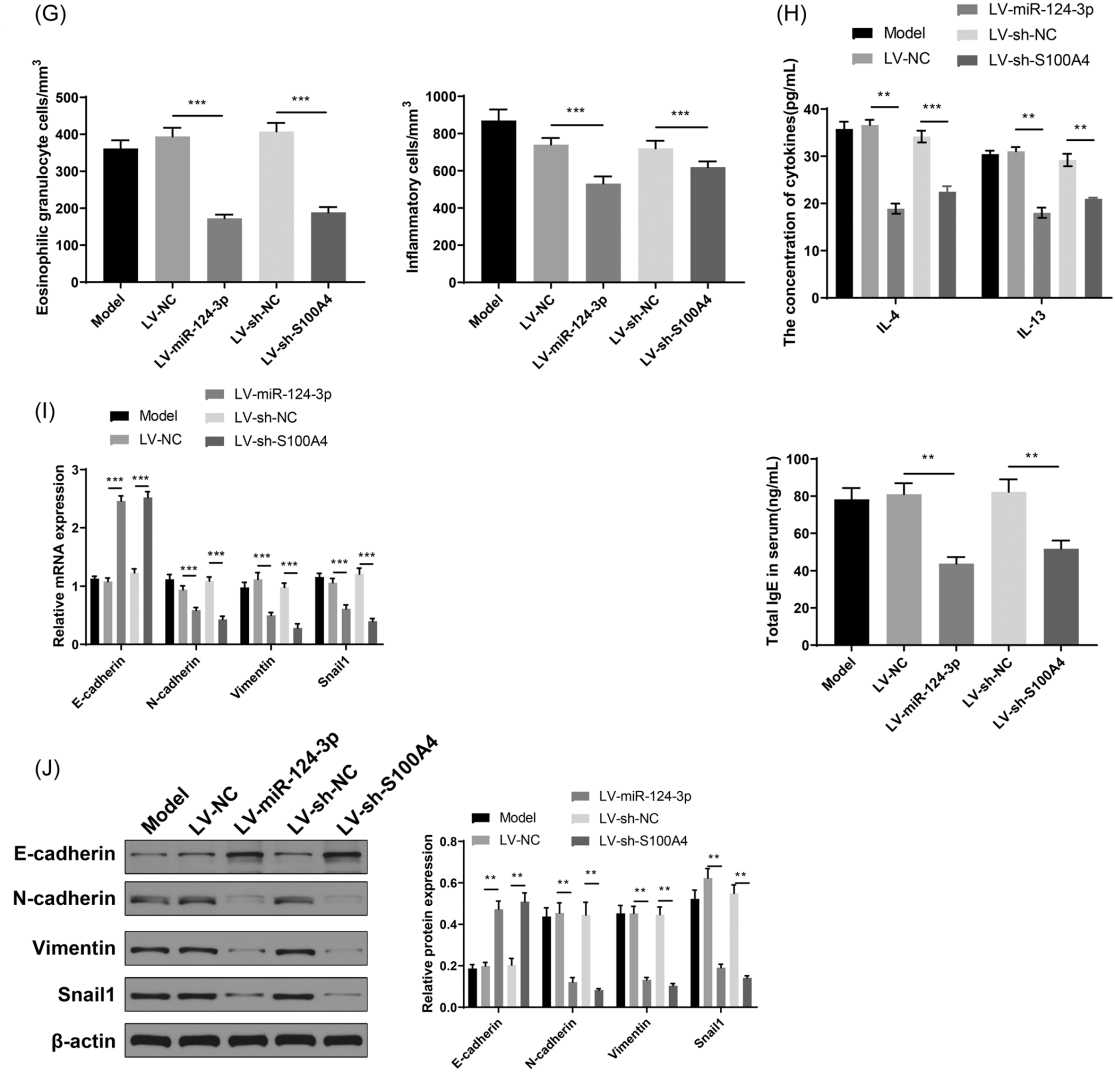

### 3.3 miR‐124‐3p targets and negatively regulates S100A4 expression

**Figure 4:**
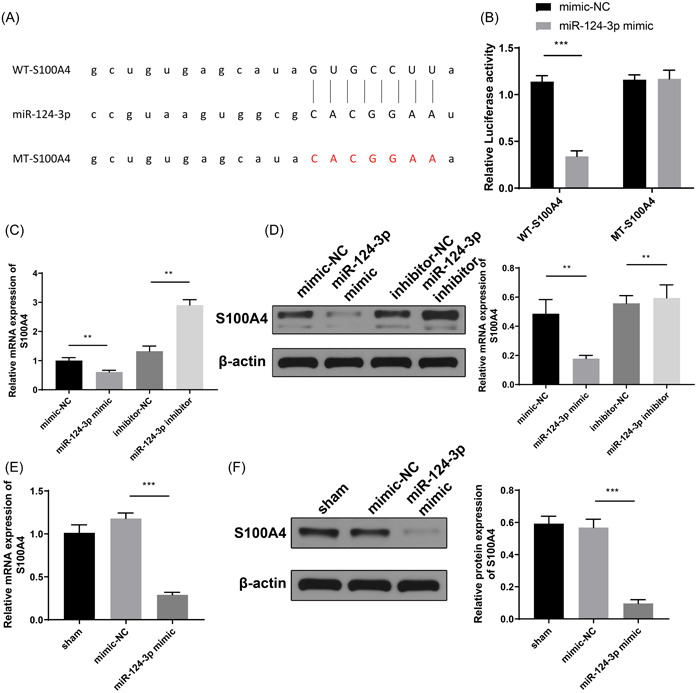
miR‐124‐3p negatively regulates S100A4 expression. (A) Starbase database predicted the binding sites of miR‐124‐3p with S100A4 and the mutant sites were accordingly designed. (B) The interaction between miR‐124‐3p and S100A4 was determined by dual‐luciferase reporter gene assay; RT‐qPCR (C) and western blot analysis (D) were conducted to assess the expression levels of S100A4 mRNA and protein. RT‐qPCR (E) and western blot analysis (F) were carried out to test the expression levels of S100A4 mRNA and protein in asthmatic mouse models. **p < .01, ***p < .001. All experiments were conducted for three times and triplicate wells were set for each experiment. RT‐qPCR, reverse transcription‐quantitative polymerase chain reaction.

StarBase was used to predict the binding sites between miR‐124‐3p and S100A4 (Figure 4A). Luciferase activity in BEAS‐2B cells, after cotransfection with WT‐S100A4 and miR‐124‐3p mimic, was suppressed (*p* < .01 vs. mimic‐NC group). In cells cotransfected with MT‐S100A4 and mimic‐NC/miR‐124‐3p, the luciferase activity was not significantly changed (Figure 4B, *p* > .05). BEAS‐2B cells were transfected with an miR‐124‐3p inhibitor or miR‐124‐3p mimic before detecting the expression levels of S100A4. The miR‐124‐3p mimic group showed suppressed S100A4 expression, whereas the miR‐124‐3p inhibitor group showed increased expression of S100A4 in BEAS‐2B cells (Figure 4C,D, *p* < .05, vs. mimic‐NC group or inhibitor‐NC group). Further detection showed that the expression of S100A4 in lung tissues of mice in the miR‐124‐3p mimic group was suppressed when compared with that in the mimic‐NC group (Figure 4E,F, *p* < .05).

### 3.4 miR‐124‐3p targets S100A4 to regulate the TGF‐β1/Smad2 signaling pathway

**Figure 5:**
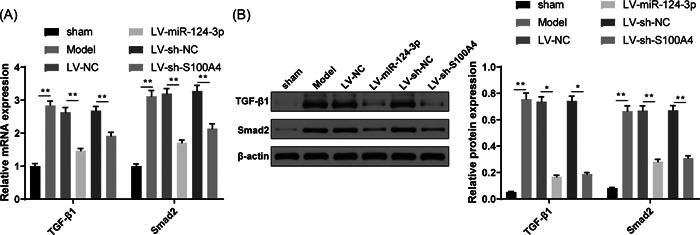
miR‐124‐3p suppresses the TGF‐β1/Smad2 signal pathway in asthmatic mouse models. RT‐qPCR (A) and western blot analysis (B) were performed to test the expression levels of TGF‐β1 and Smad2 in asthmatic mouse models. *p < .05, **p < .01. Each group had six mice and all experiments were conducted three times. RT‐qPCR, reverse transcription‐quantitative polymerase chain reaction.

RT‐qPCR and western blot analysis showed that the expression levels of TGF‐β1 and Smad2 in lung tissues of mice in the model group were elevated (Figure 5A,B, *p* < .01, vs. sham group), indicating that asthma can activate the TGF‐β1/Smad2 signaling pathway. The expression levels of TGF‐β1 and Smad2 in mice of the LV‐miR‐124‐3p and LV‐sh‐S100A4 groups were suppressed (Figure 5A,B, *p* < .01, vs. model group).

## 4 DISCUSSION

Studies based on rodents and humans have provided crucial ways to improve our understanding of pathology and identify potential associated mechanisms. The results of this study highlighted the fact that miR‐124‐3p is associated with EMT and asthma progression and functions by suppressing the activation of the TGF‐β1/Smad2 signaling pathway by negatively regulating S100A4. Evidence in this study suggests the potential implications of miR‐124‐3p and S100A4 in asthma progression and the potential roles of these two factors in targeted therapy for asthma. After establishing asthma models in rodents, we measured the expression levels of miR‐124‐3p and S100A4 and found downregulated expression of miR‐124‐3p and upregulated S100A4 expression. Similar evidence has been found in previous studies reporting the implications of S100A4^23^ and miR‐124‐3p^24^ in inflammatory diseases, including hepatic inflammation, steatosis, and inflammation‐associated tumorigenesis. Consistently, miR‐124‐3p has been reported to suppress neuronal inflammation and therefore contribute to neurite outgrowth by targeting PDE4B and inhibiting mTOR signaling or by targeting STAT3.^25^ Concerning respiratory diseases, miR‐124‐3p was discovered to be decreased in the lungs of mouse models and was found to directly target p65 to attenuate the inflammatory response and pulmonary injury.^26^ In multifactorial inflammatory diseases, such as ulcerative colitis, miR‐124‐3p might also regulate cellular apoptosis and reactive oxygen species production.^27^

S100A4 is a protein that is well‐known for its involvement in pulmonary diseases. For example, the number of S100A4^+^ macrophages in BALF has been shown to correlate with the initiation of idiopathic pulmonary fibrosis.^25^ Additionally, increased S100A4 expression has been observed in an allergen‐induced mouse asthma model.^18^ S100A4, also known as fibroblast‐specific protein 1, expressed in the basal epithelium, is an established marker of EMT based on oncology studies.^28^, ^29^, ^30^ In our study, elevated S100A4 expression was observed in asthmatic mouse models. Moreover, the overexpression of miR‐124‐3p or knockdown of S100A4 was found to regulate inflammatory cytokines and EMT, thus attenuating asthma progression, indicating the involvement of miR‐124‐3p and S100A4 in asthma. In asthma, airway inflammation requires the synthesis and secretion of inflammatory cytokines by the airway smooth muscle tissues.^31^ The release of these cytokines could enhance airway remodeling by increasing cell proliferation or mucus production.^32^ EMT is a process in which epithelial cells lose their epithelial proteins (e.g., E‐Cadherin) that are responsible for tight junctions. The cells tend to move to a more mesenchymal phenotype when they gain the mesenchymal markers, including N‐Cadherin, Vimentin, and Fibronectin, as well as the Snail, Slug, and Twist (fibroblast proliferation transcription factors).^7^ As reported, cough‐variant asthma in vitro models leads to upregulation of N‐cadherin, α‐SMA, and vimentin, and downregulation of E‐cadherin in BEAS‐2B cells.^33^ Accordingly, asthma mice had suppressed expression of E‐cadherin and increased expression of N‐cadherin, Snail1, and vimentin in our research. IgE plays a central part in asthma and other allergic diseases, which is generally elevated in patients with asthma.^34^ Marinho et al.^35^ have supported that rhinitis/rhinoconjunctivitis elevates with the increasing IgE level. Moreover, the incidence of asthma is higher when the IgE level is increased.^36^ In our study, we also found an elevated level of IgE level, which was in accord with this finding.

The dual‐luciferase reporter gene assay showed that S100A4 was a downstream target of miR‐124‐3p. A measurement of the TGF‐β1/Smad2 signaling pathway also showed that this pathway is activated in asthmatic mouse models. This study also verified the regulatory effect of miR‐124‐3p and S100A4 on the TGF‐β1/Smad2 signaling pathway, evidenced by suppressed TGF‐β1 and Smad2 expression in response to the overexpression of miR‐124‐3p or S100A4 knockdown. TGF‐β and Smad can induce the expression of α‐SMA, a myofibroblast marker.^37^ The TGF‐β1/Smad2 signaling pathway was previously reported to be involved in asthma‐related fibroblast‐to‐myofibroblast transition.^38^ Additionally, a study in China showed that in rodent asthma models, TGF‐β1/SMAD and Jagged1/Notch1 signaling pathways are regulated by recombinant pyrin domain protein to suppress asthmatic airway inflammation and airway remodeling.^39^

There were some limitations to this study that should be considered when interoperating the results. First, this study only explored the function of miR‐124‐3p and its interaction with S100A4 in asthmatic inflammation and EMT. Considering that the pathogenesis of this disease has not yet been determined, more studies and experiments are required to identify the upstream and downstream targets of the miR‐124‐3p/S100A4 axis in asthma. Second, in this study, we only focused on asthmatic inflammation and EMT, and thus, more experiments are needed to confirm the function of the miR‐124‐3p/S100A4 axis in asthma progression, including fibrosis and oxidative stress.

Owing to the significant medical advances in the past few decades, a greater understanding of the etiology and pathogenesis of asthma has been gained, although obtaining a full understanding of this disease is still in progress. This study identified the function of miR‐124‐3p in asthma progression. Our findings may be useful for the advancement of the clinical application of miRNAs in asthma treatment, and miR‐124‐3p may be a novel potential therapeutic target in asthma. Specifically, the overexpression of miR‐124‐3p can decrease the expression of S100A4 to suppress activation of the TGF‐β1/smad2 signaling pathway. Although this current research as yet offers an incomplete picture, miR‐124‐3p deserves further attention as a practical and valid clinical biomarker.

## AUTHOR CONTRIBUTIONS

Min Liu, Xiaojiang Wang, and Shuang Liu conceived the conceptualization and were responsible for the development or design of the methodology. Min Liu, Chenghong Li, and Shi Chen provided critical materials. Min Liu and Shuang Liu performed the experiments. Min Liu, Xiaojiang Wang, and Fajiu Li analyzed the data. Fajiu Li, Shi Chen, and Xiaoyan Gao wrote and edited the manuscript.

## CONFLICT OF INTEREST

The authors declare no conflict of interest.

## ETHICS STATEMENT

The design of the experiment was approved by the ethical committee of Affiliated Hospital of Jianghan University. The experiments were performed according to the ARRIVE guidelines.

## Data Availability

The datasets used or analyzed during the current study are available from the corresponding author on reasonable request.

## References

[1] Pignataro FS , Bonini M , Forgione A , Melandri S , Usmani OS . Asthma and gender: the female lung. Pharmacol Res. 2017;119:384‐390. 28238829 10.1016/j.phrs.2017.02.017

[2] Mishra V , Banga J , Silveyra P . Oxidative stress and cellular pathways of asthma and inflammation: therapeutic strategies and pharmacological targets. Pharmacol Ther. 2018;181:169‐182. 28842273 10.1016/j.pharmthera.2017.08.011 PMC5743757

[3] Lambrecht BN , Hammad H . The airway epithelium in asthma. Nat Med. 2012;18:684‐692. 22561832 10.1038/nm.2737

[4] Banno A , Reddy AT , Lakshmi SP , Reddy RC . Bidirectional interaction of airway epithelial remodeling and inflammation in asthma. Clin Sci. 2020;134:1063‐1079. 10.1042/CS20191309 32369100

[5] Fehrenbach H , Wagner C , Wegmann M . Airway remodeling in asthma: what really matters. Cell Tissue Res. 2017;367:551‐569. 28190087 10.1007/s00441-016-2566-8 PMC5320023

[6] Liu T , Liu Y , Miller M , et al. Autophagy plays a role in FSTL1‐induced epithelial mesenchymal transition and airway remodeling in asthma. Am J Physiol Lung Cell Mol Physiol. 2017;313:L27‐L40. 28473327 10.1152/ajplung.00510.2016

[7] Rout‐Pitt N , Farrow N , Parsons D , Donnelley M . Epithelial mesenchymal transition (EMT): a universal process in lung diseases with implications for cystic fibrosis pathophysiology. Respir Res. 2018;19:136. 30021582 10.1186/s12931-018-0834-8 PMC6052671

[8] Hackett TL . Epithelial‐mesenchymal transition in the pathophysiology of airway remodelling in asthma. Curr Opin Allergy Clin Immunol. 2012;12:53‐59. 22217512 10.1097/ACI.0b013e32834ec6eb

[9] Roffel MP , Bracke KR , Heijink IH , Maes T . miR‐223: A key regulator in the innate immune response in asthma and COPD. Front Med. 2020;7:196. 10.3389/fmed.2020.00196 PMC7249736 32509795

[10] Yu LL , Zhu M , Huang Y , et al. Metformin relieves acute respiratory distress syndrome by reducing miR‐138 expression. Eur Rev Med Pharmacol Sci. 2018;22:5355‐5363. 30178862 10.26355/eurrev_201808_15737

[11] Li QC , Liang Y , Su ZB . Prophylactic treatment with MSC‐derived exosomes attenuates traumatic acute lung injury in rats. Am J Physiol Lung Cell Mol Physiol. 2019;316:L1107‐L1117. 30892077 10.1152/ajplung.00391.2018

[12] Zhang W , Xue F , Xie S , Chen C , Li J , Zhu X . Isoflurane promotes proliferation of squamous cervical cancer cells through mTOR‐histone deacetylase 6 pathway. Mol Cell Biochem. 2020;476(1):45‐55. 32833118 10.1007/s11010-020-03884-7 PMC7867516

[13] Liu Y , Li L , Song X . Exosomal circPVT1 derived from lung cancer promotes the progression of lung cancer by targeting miR‐124‐3p/EZH2 axis and regulating macrophage polarization. Cell Cycle. 2022;21:514‐530. 35025697 10.1080/15384101.2021.2024997 PMC8942510

[14] Li PC , Tu MJ , Ho PY , et al. In vivo fermentation production of humanized noncoding RNAs carrying payload miRNAs for targeted anticancer therapy. Theranostics. 2021;11:4858‐4871. 33754032 10.7150/thno.56596 PMC7978307

[15] Liu Q , Shen Y , Xiao Y , et al. Increased miR‐124‐3p alleviates type 2 inflammatory response in allergic rhinitis via IL‐4Ralpha. Inflamm Res. 2022;71:1271‐1282. 35922673 10.1007/s00011-022-01614-x PMC9616750

[16] Zhang S , Dong D , Zhang Y , Wang J , Liu L , Zhao Y . miR‐124‐3p relieves allergic rhinitis by inhibiting dipeptidyl peptidase‐4. Int Immunopharmacol. 2021;101:108279. 34715574 10.1016/j.intimp.2021.108279

[17] Li Z , Li Y , Liu S , Qin Z . Extracellular S100A4 as a key player in fibrotic diseases. J Cell Mol Med. 2020;24:5973‐5983. 32307910 10.1111/jcmm.15259 PMC7294136

[18] Huang X , Qu D , Liang Y , Huang Q , Li M , Hou C . Elevated S100A4 in asthmatics and an allergen‐induced mouse asthma model. J Cell Biochem. 2019;120:9667‐9676. 30569582 10.1002/jcb.28245

[19] Choe N , Kwon DH , Shin S , et al. The microRNA miR‐124 inhibits vascular smooth muscle cell proliferation by targeting S100 calcium‐binding protein A4 (S100A4). FEBS Lett. 2017;591:1041‐1052. 28235243 10.1002/1873-3468.12606

[20] Lee BW , Ha JH , Ji Y , et al. Alnus hirsuta (Spach) Rupr. attenuates airway inflammation and mucus overproduction in a murine model of ovalbumin‐challenged asthma. Front Pharmacol. 2021;12:614442. 33643046 10.3389/fphar.2021.614442 PMC7902870

[21] Wang Z , Ji N , Chen Z , et al. MiR‐1165‐3p suppresses Th2 differentiation via targeting IL‐13 and PPM1A in a mouse model of allergic airway inflammation. Allergy Asthma Immunol Res. 2020;12:859‐876. 32638565 10.4168/aair.2020.12.5.859 PMC7346992

[22] Dong L , Wang Y , Zheng T , et al. Hypoxic hUCMSC‐derived extracellular vesicles attenuate allergic airway inflammation and airway remodeling in chronic asthma mice. Stem Cell Res Ther. 2021;12:4. 33407872 10.1186/s13287-020-02072-0 PMC7789736

[23] Reyes M , González L , Ibeas K , et al. White adipose tissue‐infiltrated CD11b+ myeloid cells are a source of S100A4, a new potential marker of hepatic damage. Eur J Endocrinol. 2021;184:533‐541. 33524007 10.1530/EJE-20-1130

[24] Murray‐Stewart T , Sierra JC , Piazuelo MB , et al. Epigenetic silencing of miR‐124 prevents spermine oxidase regulation: implications for *Helicobacter pylori*‐induced gastric cancer. Oncogene. 2016;35:5480‐5488. 27041578 10.1038/onc.2016.91 PMC5050049

[25] Huang S , Ge X , Yu J , et al. Increased miR‐124‐3p in microglial exosomes following traumatic brain injury inhibits neuronal inflammation and contributes to neurite outgrowth via their transfer into neurons. FASEB J. 2018;32:512‐528. 28935818 10.1096/fj.201700673R

[26] Liang Y , Xie J , Che D , et al. MiR‐124‐3p helps to protect against acute respiratory distress syndrome by targeting p65. Biosci Rep. 2020;40:BSR20192132. 32391561 10.1042/BSR20192132 PMC7253404

[27] Luo Y , Yu MH , Yan YR , et al. Rab27A promotes cellular apoptosis and ROS production by regulating the miRNA‐124‐3p/STAT3/RelA signalling pathway in ulcerative colitis. J Cell Mol Med. 2020;24:11330‐11342. 32815642 10.1111/jcmm.15726 PMC7576264

[28] Kalluri R , Weinberg RA . The basics of epithelial‐mesenchymal transition. J Clin Invest. 2009;119:1420‐1428. 19487818 10.1172/JCI39104 PMC2689101

[29] Tochimoto M , Oguri Y , Hashimura M , et al. S100A4/non‐muscle myosin II signaling regulates epithelial‐mesenchymal transition and stemness in uterine carcinosarcoma. Lab Invest. 2020;100:682‐695. 31857700 10.1038/s41374-019-0359-x

[30] Zeisberg M , Neilson EG . Biomarkers for epithelial‐mesenchymal transitions. J Clin Invest. 2009;119:1429‐1437. 19487819 10.1172/JCI36183 PMC2689132

[31] Wu Y , Zhang W , Gunst SJ . S100A4 is secreted by airway smooth muscle tissues and activates inflammatory signaling pathways via receptors for advanced glycation end products. Am J Physiol Lung Cell Mol Physiol. 2020;319:L185‐L195. 32432920 10.1152/ajplung.00347.2019 PMC7468847

[32] Hu L , Li L , Zhang H , et al. Inhibition of airway remodeling and inflammatory response by Icariin in asthma. BMC Complement Altern Med. 2019;19:316. 31744482 10.1186/s12906-019-2743-x PMC6862818

[33] Si Z , Zhang B . Amygdalin attenuates airway epithelium apoptosis, inflammation, and epithelial‐mesenchymal transition through restraining the TLR4/NF‐κB signaling pathway on LPS‐treated BEAS‐2B bronchial epithelial cells. Int Arch Allergy Immunol. 2021;182:997‐1007. 34428767 10.1159/000514209

[34] Kim JH , Cheong HS , Park JS , et al. A genome‐wide association study of total serum and mite‐specific IgEs in asthma patients. PLoS One. 2013;8:e71958. 23967269 10.1371/journal.pone.0071958 PMC3742455

[35] Marinho S , Simpson A , Söderström L , Woodcock A , Ahlstedt S , Custovic A . Quantification of atopy and the probability of rhinitis in preschool children: a population‐based birth cohort study. Allergy. 2007;62:1379‐1386. 17822449 10.1111/j.1398-9995.2007.01502.x

[36] Cui L , Yin J . Association of serum specific IgE levels with asthma in autumn pollen‐induced allergic rhinitis: a retrospective analysis. J Asthma. 2019;56:505‐511. 29667465 10.1080/02770903.2018.1466316

[37] Hu B , Wu Z , Phan SH . Smad3 mediates transforming growth factor‐β‐Induced α‐Smooth muscle actin expression. Am J Respir Cell Mol Biol. 2003;29:397‐404. 12702545 10.1165/rcmb.2003-0063OC

[38] Paw M , Wnuk D , Kadziolka D , et al. Fenofibrate reduces the asthma‐related fibroblast‐to‐myofibroblast transition by TGF‐Beta/Smad2/3 signaling attenuation and Connexin 43‐dependent phenotype destabilization. Int J Mol Sci. 2018;19:2571. 30158495 10.3390/ijms19092571 PMC6163263

[39] Wang Z , Jiang J , Wang C , et al. [Recombinant pyrin domain protein attenuates airway inflammation and airway remodeling through TGF‐β1/SMAD and Jagged1/Notch1 signaling pathways in chronic bronchial asthma mice]. Xi Bao Yu Fen Zi Mian Yi Xue Za Zhi. 2020;36:220‐227. 32389169

